# Smoke-Free Medical Facility Campus Legislation: Support, Resistance, Difficulties and Cost

**DOI:** 10.3390/ijerph6010246

**Published:** 2009-01-13

**Authors:** Christine Sheffer, Maxine Stitzer, J. Gary Wheeler

**Affiliations:** 1 Health Behavior and Health Education Department, College of Public Health, University of Arkansas for Medical Sciences, 4301 West Markham St #820, Little Rock, AR 72205, U.S.A; 2 Psychiatry and Behavioral Sciences, Johns Hopkins University School of Medicine, Johns Hopkins Bayview Medical Center, 5510 Nathan Shock, U.S.A.; E-mail: mstitzer@jhmi.edu (M. S.); 3 Department of Pediatrics, College of Medicine, University of Arkansas for Medical Sciences, 4301 West Markham St Little Rock, AR 72205, U.S.A.; E-mail: wheelergary@uams.edu (J. G. W.)

**Keywords:** Smoke-free hospitals, public smoking bans, secondhand tobacco smoke, tobacco smoking, health effects, legislated policy change

## Abstract

Although medical facilities restrict smoking inside, many people continue to smoke outside, creating problems with second-hand smoke, litter, fire risks, and negative role modeling. In 2005, Arkansas passed legislation prohibiting smoking on medical facility campuses. Hospital administrators (N=113) were surveyed pre- and post-implementation. Administrators reported more support and less difficulty than anticipated. Actual cost was 10–50% of anticipated cost. Few negative effects and numerous positive effects on employee performance and retention were reported. The results may be of interest to hospital administrators and demonstrate that state legislation can play a positive role in facilitating broad health-related policy change.

## 1. Introduction

Smoking, which causes over 438,000 deaths and $167 billion in costs annually, is the greatest source of preventable death and disease in the U.S. [1–3]. Smoke-free policies are an important component of an ecological and social-cognitive approach to reducing tobacco use and tobacco-related disease [4–6]. Nonetheless, the U.S. healthcare system has been slow to respond with comprehensive tobacco control policies [7]. Healthcare facilities serve as employers, healthcare providers, and community leaders and thus have greater responsibility than most in protecting people from tobacco smoke [8, 9]. Effective December 31, 1993, the Joint Commission on Accreditation of Health Organizations (JCAHO) introduced indoor restrictions on smoking as a quality indicator [10]. However, these JCAHO requirements *did not* restrict smoking outside facilities. One year later, 96% of hospitals were in compliance with the indoor restrictions, but only 2.7% reported smoke-free campus policies [11].

As employers, healthcare providers, and community leaders, healthcare facilities seek to provide a safe, healthy, and cost-effective environment. Employee smoking increases costs from employee illness and absenteeism as well as increased property damage, maintenance, and insurance costs [12]. Every employee who smokes costs employers approximately $3,200 in additional healthcare costs and lost productivity every year [13–16]. Patient smoking negatively affects many medical treatments and procedures, inhibits bone and wound healing, and doubles the risk of post-operative infection [3, 17]. Although the JCAHO mandate made smoking more inconvenient, at least 25% of smokers report smoking while in the hospital with 82–90% of outdoor smoking clustered within 10 meters of building entrances, exposing others to second-hand smoke and creating problems with litter, fire risk, and negative role modeling in highly trafficked and visible areas [18–20]. Patients are often more vulnerable to the effects of second-hand smoke, and if quitting, can benefit from smoke-free policies [8, 20]. Designated outdoor smoking areas fail to address these issues, especially when nearly 50% of on-campus, outdoor smokers are employees [19, 20]. A smoke-free campus models healthy behavior and sends a clear message that the facility supports the health of employees, patients, and the community. Establishing smoke-free campuses provides leadership in this domain, influences the community’s attitudes toward tobacco use, encourages and facilitates cessation, and has been shown to cause a significant reduction in employee smoking [7, 12, 14, 21, 22]. A smoke-free campus can provide a safer, healthier, and more cost-effective medical environment.

While few severe problems are actually reported with smoke-free policies, a number of concerns remain about a lack of patient and visitor acceptance and negative employee morale [7, 11, 23, 24]. Some concerns have been expressed anecdotally and in letters to editors suggesting that smoke-free campus policies are impractical and unethical because smokers will be unable to refrain from smoking; are destined to smoke in uncontrolled areas; will be forced to compromise their treatment by leaving the grounds to smoke; and by doing so will aggravate existing distress while subjugating patient needs in favor of a policy [18, 25–27]. Consequently, administrators considering smoke-free campus policies are often unsure of the support and the resistance they will experience from employees, patients, visitors, physicians, the board, and the community; concerned about the prospect of losing good employees; and concerned about being financially penalized as smokers seek facilities on whose grounds they can smoke [7, 11, 23, 24].

In order to address these issues, the Arkansas state legislature passed Act 134 in March of 2005. This was groundbreaking legislation that prohibited smoking in all the “buildings and property in and on which the medical facility operates together with all property owned that is contiguous to the buildings in which medical services are provided.” This legislation was enacted primarily because the Arkansas Hospital Association (AHA) supported it. The AHA supported the legislation because a number of facilities were already planning to enact smoke-free campus policies and implementation of a unilateral, comprehensive, statewide medical facility campus smoking ban would enable facilities to implement smoke-free campus policies without being unduly penalized by market forces [7]. Psychiatric and alcohol and drug (A&D) facilities, however, opposed the legislation because representatives felt that these patients would be negatively affected, and were thus exempted as were federally controlled facilities. Act 134 became effective October 1, 2005. Once the legislation was passed, the University of Arkansas for Medical Sciences College of Public Health supported the AHA by compiling a Smoke-Free Hospital Toolkit comprised of a booklet to guide implementation and a resource CD. The toolkit was theoretically grounded in ecological and social cognitive perspectives, but also utilized experiential and empirical sources to guide development [7–9, 24]. Numerous written resources were provided on the CD including administrative and clinical guidelines, examples of policy statements, signage, training activities, and problem-solving. The toolkit is available as a download at (http://www.uams.edu/coph/reports/SmokeFree_Toolkit/). A hard copy was distributed to the AHA membership by AHA.

The aim of this study was to characterize the perceived concerns and sources of support and resistance reported by the Chief Executive Officers (CEOs) and administrators of Arkansas medical facilities before and after Act 134 became effective. Interviews were conducted with the same facilities both before and after the effective date. Information was collected to identify significant sources of support and resistance while considering the implementation of smoke-free hospital campus policies. Pre-implementation variables were also used to predict progress with establishing a completely smoke-free campus 12 months after Act 134 took effect. This investigation also provides evaluative information on the first legislated, statewide prohibition of smoking on medical facility grounds.

## 2. Method

### 2.1. Participants

This study was approved by the Institutional Review Board at the University of Arkansas for Medical Sciences. A list of 110 member medical facilities and CEO/administrators was obtained from the AHA. Three additional facilities were subsequently identified through contact with hospital CEOs for a total of 113. Information about number of hospital beds, psychiatric and/or alcohol and drug (A&D) beds, and financial control status (private non-profit, city, state, federal, county, or corporate) was obtained from the AHA. The number of beds at the AHA member medical facilities ranged from 0 to 791, with a mean of 132, a median of 77, and a mode of 25. The majority of facilities had no psychiatric or A&D beds (n=68; 64.76%), with 27.62% (n=29) maintaining some psychiatric and A&D beds, and 7.62% (n=8) maintaining only psychiatric and/or A&D beds. The majority of medical facilities were private non-profit (56.36%), with 26.36% under corporate control, and 17.27% under city, county, state, or federal government control.

### 2.2. Survey Instrument

The instrument used for pre- and post-implementation data collection was a 23 item questionnaire delivered over the telephone assessing the degree to which CEO/administrators (a) perceived their facility to have completed implementation of the new policy; (b) were in favor of or “agreed” with the legislation; (c) anticipated/experienced support from employees, patients, visitors, physicians, the board, and the community; and (d) anticipated/experienced resistance from employees, patients, visitors, physicians, the board, and the community. Responses to questions (a) through (d) were made on an 11 point discrete analogue scale where 0 = “have not started,” “none at all” or “do not agree at all,” and 10 = “process complete,” “the most possible,” or “total agreement.” Several open-ended questions were also included in the pre- and post-implementation surveys concerning (e) anticipated/experienced cost of implementation; (f) the greatest challenges for implementation; (g) negative effects of the policy on employee performance and retention, and (h) positive effects of the policy on employee performance and retention. Post-implementation, respondents were also asked to identify helpful resources and discuss the helpfulness of the Smoke-free Hospital Toolkit.

### 2.3. Procedure

During the April and May 2005 monthly meetings of the AHA, the Smoke-free Hospital Toolkit was distributed to all members and the CEO/administrators were asked to respond to an upcoming telephone survey concerning Act 134. The pre-implementation survey was administered during April/May 2005 to CEO/administrators at all the medical facilities. The post-implementation survey was administered in October 2006, 16 months after Act 134 was passed and 12 months after Act 134 became effective.

### 2.4. Analysis

Data were entered into a database and analyzed using SPSS version 12 [28]. Descriptive analyses were conducted on all variables. Progress, agreement, support, and resistance items were analyzed with a paired samples t-tests (alpha < 0.05). Open-ended responses were categorized and summarized by similar words, meanings, and/or themes.

Analyses of variance (ANOVA) were utilized to test for significant differences among the non-exempt government controlled (county, city, state), private non-profit, and the corporate controlled facilities on the completion, support, and resistance variables (a through d above) both pre- and post-implementation, followed by Bonferroni *post hoc* tests to identify significant differences and control for Type I family-wise error.

A series of backward stepwise multiple regression analyses were conducted to develop a parsimonious model that predicted progress toward implementation 12 months after Act 134 look effect. The predictors included: 1) number of beds; 2) number of psychiatric and A&D beds; 3) financial control (government, private non-profit, corporate controlled); pre-implementation agreement as an 4) employer, 5) healthcare provider, and 6) community leader; pre-implementation level of support from the 7) employees, 8) patients, 9) visitors, 10) board, 11) physicians, 12) community; and pre-implementation resistance from 13) employees, 14) patients, 15) visitors, 16) board, 17) physicians, 18) community. The criterion probability of F for a variable to be removed from the model was >/= 0.10.

## 3. Results

### 3.1. Response Rates

The survey required approximately 10 minutes to administer; however, several respondents provided lengthy comments post-implementation that extended the interview. The longest interview required 35 minutes to complete.

The initial survey administration revealed some confusion on the part of the facilities as to whether the legislation applied to their facility or not. Of the 113 facilities contacted for the pre-implementation survey, 99 (87.61%) responded. Of the 99 respondents, 11 correctly did not consider the legislation to apply to their facility because they were located out of state, maintained only psychiatric and/or A&D beds, or were federally controlled. However, four *incorrectly* assumed that the legislation did not apply because the facility leased space, leaving 84 respondents who completed a survey.

The confusion about the applicability of the legislation appeared to have been resolved 12 months after the legislation became effective. Of the original 113 facilities, 78 (69.02%) responded to the post-implementation survey, of which eight correctly assumed that the legislation did not apply because they were located out of state or maintained only psychiatric and/or A&D beds. Two facilities reported that they had closed, leaving 68 respondents who completed the survey. The respondents to the post-implementation survey included 18 facilities that did not respond to the pre-implementation survey (14 non-respondents and four facilities that initially incorrectly assumed that the legislation did not apply to them). Thus, paired responses were obtained from 50 facilities, 44.25% of the original 113 facilities.

There were no significant differences in number of beds, financial control status, and the presence of psychiatric and/or A&D beds between facilities that responded to both surveys and facilities that did not (F(1,103)=1.98, p=.16; χ^2^(5, N=110) = 2.29, p=0.81; χ^2^(2, N=105) = 2.86, p=0.24). There was also no significant difference between facilities that responded to both surveys and facilities that did not in terms of level of agreement with the legislation as an employer, healthcare provider, or community member (F(1,73)=0.041, p=0.84; F(1,73)=.81, p=0.37; F(1,73)=.24, p=0.63).

### 3.2. Levels of Progress, Agreement, Support, and Resistance

As shown in Table 1, item 1, the facilities clearly made significant progress with implementation. CEO/administrator level of agreement with the legislation was high both pre- and post-implementation with no significant change over time. Facilities experienced significantly more support than anticipated from employees, patients, the board, and the physicians; and significantly less resistance than anticipated from employees, visitors, and the board.

### 3.3. Estimated Cost

Pre-implementation, only 37 facilities were willing or able to estimate the projected cost for implementation. Pre-implementation projected costs ranged from $200 to $150,000 with a mean of $19,620 (SD $37,425), a median of $5,000, and a mode of $5,000. Post-implementation cost estimates were obtained from 55 facilities and ranged from $10 to $60,000 with a mean of $6,447 (SD $12,724), a median of $1,000, and a mode of $1,000.

Responses from the facilities which provided paired pre- and post-implementation responses (n=22) also indicate that the actual cost of implementation was much less than expected. Pre-implementation, the mean estimated cost was $14,020 (SD = $31,356), median $5,500, and mode was $10,000. Post-implementation, the mean estimated cost was $6,209 (SD=$12,483) with a median of $1,500, and a mode of $1,000. The mean actual cost was 44% of the anticipated mean cost; the median cost was 27% of the anticipated median cost; and the modal cost was 10% of the anticipated modal cost.

### 3.4. Greatest Challenges

When facilities were asked to anticipate their greatest challenges pre- and post-implementation, the pre-implementation responses were concise, often comprised of one or two words and most often mentioned communication and/or education about and enforcement. However, post-implementation, the respondents clearly demonstrated experience with the process and provided numerous details about their challenges with enforcement, communication and education. See Table 2 for details.

### 3.5. Most Helpful

Post-implementation, 68 facilities responded to the question about what was most helpful in the process, the answers included practical assistance such as, “The toolkit was helpful,” and elements in the toolkit such as “Getting policies from other hospitals.” Governmental or organizational support was cited such as, “The legislation itself,” and “That it was a law, took the pressure off the hospitals as individuals,” and “the support of the Arkansas Hospital Association.” Also cited was the cooperation of smoking employees: “We have actually had positive support from smokers, agreeing that smoking doesn’t belong in hospitals” and the support of non-smokers: “Non-smoking employees like the plan because their counterparts are no longer taking extra smoke breaks. It creates a more level playing field.”

### 3.6. Effect on Employee Retention, Attitudes, and Smoking Behaviors

The overall effect of Act 134 on employees appeared positive. See Tables 3 and 4 for details.

### 3.7. Differences among Government Controlled, Private Non-Profit, and Corporate Controlled Facilities

Pre-implementation, there were no significant differences among government controlled, private non-profit, and corporate controlled facilities on the degree of progress made toward implementation, level of agreement, or the support and the resistance anticipated from employees, patients, visitors, the board, or physicians. Post-implementation, a significant difference was found between government controlled (M = 5.50) and corporate controlled (M=7.30) facilities on the level of support experienced from patients (F (2, 61) = 3.8, p.=0.028). Corporate controlled facilities experienced significantly more support from patients than government controlled facilities.

### 3.8 Predicting Progress with Implementation

The degree to which facilities had made progress toward establishing a completely smoke-free campus 12 months after Act 134 took effect was parsimoniously predicted by five of the 18 variables: 1) fewer psychiatric and A&D beds; 2) less anticipated agreement with the law as a healthcare provider; 3) less anticipated support from visitors; 4) more anticipated support from the community; and 5) less anticipated resistance from physicians (R^2^= .25, p.=.008). See Table 5.

## 4. Discussion

This investigation provides the first quantified, experiential evidence regarding the widespread implementation of smoke-free medical facility campus policies with data collected systematically both pre- and post-implementation from a large sample of medical facilities undergoing the same process at the same time. This investigation also provides evidence that smoke-free medical campus policies can be successfully legislated on a state level. Legislation such as this allows medical facilities to implement smoke-free campus policies without incurring market-force penalties such as losing patients who smoke to facilities that allow smoking.

The subjective concerns of those who spearhead policy change are vitally important to any change process. Overall, this investigation indicates that the major concerns about implementation, although thematically consistent with some of the literature, are less troublesome than anticipated. Respondents generally were accurate in anticipating the relative types of issues they were to experience. For instance, most both foresaw and experienced their greatest challenges as communication and enforcement. However, respondents underestimated the support they were to receive from employees, patients, the board, and physicians; and overestimated the resistance from employees, visitors, and the board. Overall, there was little negative effect on employee performance and retention, and some positive effects on employees. Additionally, implementation was much less costly than anticipated.

Interestingly, among the multiple comparisons made between the types of facilities, the only significant difference found was on the level of support experienced from patients. Corporate controlled facilities reported significantly more support from patients than government controlled facilities. Speculatively, these facilities may serve different patient populations with government controlled facilities serving a larger proportion of lower socio-economic status (SES) patients. Lower SES groups smoke at nearly double the prevalence rate of higher SES groups [29]. If a facility served a larger proportion of lower SES patients, the task of communicating and enforcing the new policy to a larger proportion of patients may have been more difficult and respondents could have easily experienced less support if their patient population included more smokers.

The degree to which facilities had made progress toward compliance with Act 134 and established a completely smoke-free campus was parsimoniously predicted by five variables. Given that the Arkansas psychiatric and A&D treatment community believed that Act 134 would adversely affect these patients, it is unsurprising that the fewer psychiatric and A&D beds within a facility, the more likely the facility was to have established a completely smoke-free campus. It also makes intuitive sense that the more support from the community and the less resistance from physicians, the more likely the facility was to have established a completely smoke-free campus. Less obvious perhaps, are the reasons why less facility agreement with the law in their role as a healthcare provider and less anticipated support from visitors predicted greater progress. Perhaps facilities that were farther along in the process were less likely to consider their role as healthcare providers as important to compliance with the law and that implementation was driven by other factors. Similarly, facilities that were farther along expected less support from visitors, but may have had more realistic expectations of visitors and prepared more effectively. These facilities may have attributed their compliance to other factors that were not queried. Realistically, establishing a smoke-free campus in response to Act 134 occurred within a complex social environment for each facility with a plethora of responses to the factors contributing to progress and success.

Because this study included a large number of facilities of different types, these results are likely to be generalizable to other contexts. Support from the hospital association, however, may be a factor that mediates generalizability of these results in that it was instrumental in passing Act 134. Without AHA support, passage of the law would have been difficult. However, among individual AHA members, there was a full range in the level of agreement with the law on each of the agreement items (range 0–10). So although support from the state hospital association was key for passage, it may or may not mediate progress made toward implementation once such a law is passed or a decision to implement a policy is made. This information may be useful to state hospital associations in other states as well as smoke-free advocates interested in establishing smoke-free medical campuses. Additionally, the finding that facilities experience less difficulty than anticipated is similar to other findings [7].

Clearly, enforcement was a significant concern for the facilities as evidenced by the responses to the greatest challenges question. However, open-ended responses revealed that the majority of facilities (54.79%) reported that they experienced, “none,” “low,” “minimal,” “mild,” “not much,” “fairly little” “not a whole lot,” or “very little” difficulty with enforcement or reported that the process was “fairly easy.” One facility reported, “a great deal of difficulty;” one stated, “enforcement was hard;” two reported that the process was “very difficult” and two reported difficulties with employers and patients. These results are also consistent with other findings [7].

Summarily, many comments and responses to open-ended questions were useful for obtaining insight into the implementation process. Some unanticipated challenges were noted: “A few doctors felt people should be able to smoke, so they provided benches on their private property for the smokers;” “Die-hard smokers that are key contributors to our community, [found it] hard to make a change;” “Employees would go across the street [to smoke] and personal property house owners had to put signs up;” and “Smokers stand in the street and drivers are worried about running them over.” But creative solutions were also noted, “I decided to put up signs around the smoker hideouts that say: Danger, venomous snakes found in this area.” These comments suggest that although every facility experienced similar challenges as evidenced by the consistency of the responses to the greatest challenges question, each facility was also confronted with unique challenges and situations that required creative approaches to resolve.

There are several limitations to this study. While this study investigated the subjective concerns of the medical facility CEO’s and administrators and this focus is one of the primary strengths of the study, the subjective views were not objectively validated by observational or corroborative data. Additionally, those facilities that did not complete the pre- and post-implementation surveys may have systematically experienced more or less support or resistance than those who completed both surveys; and possibly did not achieve full implementation within the allotted time frame. Finally, the survey was confined to a single state and it is possible that hospitals in other states may have different experiences with implementing campus-wide smoke-free policies, particularly because Arkansas enacted other smoke-free legislation in July 2006, 3-months before the post-implementation survey was conducted [30].

This study illustrates the positive role that the state legislature can play in facilitating broad health-related policy change. The results address the natural, anecdotal, and empirically-based concerns expressed by many administrators and thus support further implementation of smoke-free policies at institutions such as mental health facilities, where smoke-free campus policies have yet to make substantial inroads [31].

## Figures and Tables

**Table 1.** CEO views of Act 134.

		Mean** (SD)	(n)
1. Is your campus smoke-free?	Pre	4.49 (2.92)	
	Post*	9.57 (1.08)	49
2. How much do you agree with Act 134?			
a. As an employer?	Pre	8.78 (2.38)	
	Post	9.22 (1.67)	49
b. As healthcare provider?	Pre	9.41 (1.77)	
	Post	9.80 (0.74)	49
c. As a community member?	Pre	9.10 (1.95)	
	Post	9.47 (1.26)	49
3. How much support do you anticipate/did you experience from the following:			
a. Employees?	Pre	6.86 (1.84)	
	Post*	7.68 (1.50)	50
b. Patients?	Pre	5.96 (2.41)	
	Post*	6.81 (1.88)	47
c. Visitors?	Pre	5.66 (2.26)	
	Post	6.13 (2.32)	48
d. Board?	Pre	9.42 (1.14)	
	Post*	9.84 (0.62)	50
e. Physicians?	Pre	8.94 (1.50)	
	Post*	9.54 (0.71)	50
f. Community?	Pre	7.35 (1.94)	
	Post	7.83 (2.10)	46
4. How much resistance do you anticipate/did you experience from the following:			
a. Employees?	Pre	4.62 (2.42)	
	Post*	3.64 (2.35)	50
b. Patients?	Pre	4.61 (2.46)	
	Post	4.13 (2.93)	46
c. Visitors?	Pre	5.41 (2.40)	
	Post*	4.41 (2.45)	49
d. Board?	Pre	0.40 (0.83)	
	Post*	0.02 (0.14)	50
e. Physicians?	Pre	1.10 (1.37)	
	Post	0.73 (1.40)	49
f. Community?	Pre	2.74 (1.91)	
	Post	2.00 (2.10)	46

Pre-test conducted April/May 2005, post-test October 2006;

* Post-test response was significantly different from pre-test, p < 0.05;

** All responses were on a scale of 0 to 10 where 0 = not at all and 10=most possible.

**Table 2.** Greatest challenges pre- and post-implementation*

	Pre-implementation	Post-implementation
**Greatest challenge responses**	(n=76)	(n=71)
**Enforcement**	55%	51%
**Communication and/or education**	26%	35%

* Some respondents reported more than one greatest challenge.

**Table 3.** Positive effect on employee performance and retention. How much of a positive effect do you think this policy had on employee performance and retention?

Responses	(n=65)
“Very little” or “no effect”	28%
“Very positive”	12%
“Some” or “a few” employees quit smoking	22%
A large number or “many” employees quit smoking	11%
“Improved” or “better” job performance	3%

**Table 4.** Negative effect on employee performance and retention. How much of a negative effect do you think this policy had on employee performance and retention?

Responses	(n=67)
“None” or “no effect”	63%
“Very little” or “minimal effect”	28%
“Some” or response was about one or more specific negative effects	7%

**Table 5.** Regression analysis predicting progress with implementation.

Model	B	Standard Error B	β	P value
Constant	10.79	0.597		.000
Number of beds devoted to psychiatric and alcohol and drug patients	–0.29	0.144	–0.295	.055
Facility pre-implementation agreement as a healthcare provider	–0.086	0.044	–0.300	.056
Anticipated level of support from visitors pre-implementation	–0.125	0.043	–0.541	.006
Anticipated level of support from community pre-implementation	0.178	0.060	0.592	.006
Anticipated level of resistance from physicians pre-implementation	–0.097	0.056	–0.264	.091
